# The Combined Use of Cinnamaldehyde and Vitamin C Is Beneficial for Better Carcass Character and Intestinal Health of Broilers

**DOI:** 10.3390/ijms25158396

**Published:** 2024-08-01

**Authors:** Yihong Huang, Aling Lang, Shan Yang, Muhammad Suhaib Shahid, Jianmin Yuan

**Affiliations:** State Key Laboratory of Animal Nutrition and Feeding, College of Animal Science and Technology, China Agricultural University, Beijing 100193, China; huangyh@cau.edu.cn (Y.H.); langal525@163.com (A.L.); 2023202040011@whu.edu.cn (S.Y.); suhaibvet@gmail.com (M.S.S.)

**Keywords:** cinnamaldehyde, vitamin C, growth performance, intestinal health, broiler

## Abstract

The use of cinnamaldehyde and Vitamin C can improve immunity and intestinal health. A two-way factorial design was employed to investigate the main and interactive effects of cinnamaldehyde and vitamin C on the growth, carcass, and intestinal health of broiler chickens. A total of 288 one-day-old female Arbor Acres broiler chicks were randomly distributed among four treatment groups, consisting of six replicate cages with 12 birds each. Four treatments were basal diet or control (CON), supplemental cinnamaldehyde (CA) 300 g/ton (g/t), vitamin C (VC) 300 g/t, and cinnamaldehyde 300 g/t, and vitamin C 300 g/t (CA + VC), respectively. The results showed that supplemental CA did not affect the growth performance or slaughter performance of broilers at 21 days (d), 42 days (d), and 1–42 days (d); however, it could improve intestinal barrier function at 42 d of age and reduce the mRNA expression of inflammatory factors in the intestine at 21 d and 42 d of age. Supplemental VC showed a trend towards increasing body weight gain (BWG) at 21 d (*p* = 0.094), increased breast muscle rate (at 21-d 5.33%, *p* < 0.05 and at 42-d 7.09%, *p* = 0.097), and decreased the abdominal fat (23.43%, *p* < 0.05) and drip loss (20.68%, *p* < 0.05) at 42-d. Moreover, VC improves intestinal morphology and intestinal barrier function and maintains a balanced immune response. The blend of CA and VC significantly upregulated the mRNA expression of myeloid differentiation factor 88 (*MyD-88*) in the intestine at 21 d of age, the mRNA expression of catalase (*CAT*), *Occludin*, *Claudin-1*, *Mucin-2*, nuclear factor-kappa B (*NF-κB*) and toll-like receptor 4 (*TLR-4*) in the intestine at 42 d of age (*p* < 0.01), and downregulated the mRNA expression of interleukin 10 (*IL-10*), interleukin 6 (*IL-6*), tumor necrosis factor-alpha (*TNF-α*) in the intestine at 21-d and 42-d of age, and interleukin-1 beta (*IL-1β*) mRNA in intestine at 42 d of age (*p* < 0.01). This study suggested that the combination of CA and VC had the potential to regulate intestinal health and result in better carcass character of broilers.

## 1. Introduction

The gut is the main site for digestion and absorption of nutrients, and it acts as a crucial barrier, protecting the body from harmful factors [1]. In recent years, genetic selection has been used to breed faster-growing broilers with high breeding efficiency [2] to meet the demand for chicken meat. This fast-growth genetic selection process ignores the maintenance of intestinal health, which disrupts physiological and biochemical metabolism, leading to oxidative stress, chronic inflammation, and intestinal diseases [3]. Moreover, high feeding density and feed oxidation can provoke oxidative stress, which damages intestinal health and directly decreases the broiler’s performance which increases economic losses [4,5]. Hence, it is crucial to identify a specific nutritional program that enhances the performance and intestinal health of broilers.

The new strategies involve using probiotics, prebiotics, plant extracts, and vitamins instead of antibiotics in poultry feed [6,7]. Cinnamaldehyde is an aldehyde compound that is an effective component in a variety of plant essential oils. It has antibacterial, anti-inflammatory, and anti-oxidation functions [8,9]. The addition of cinnamaldehyde to broiler diets can improve growth performance, improve gut health, and prevent feed quality deterioration [10,11]. Vitamin C (VC) is often added to the chicken diet to alleviate the adverse effects of stress on broiler production, which can enhance the body’s antioxidant system and improve immunity and intestinal health [12].

Although both cinnamaldehyde and vitamin C have the function of anti-oxidation, the efficacy of combined utilization is unknown. The previous studies use cinnamaldehyde and vitamin C alone which have certain overlap and complementary parts in promoting the performance and intestinal health of broilers [13,14]. Moreover, the combined effect of these ingredients on the performance and intestinal health of broilers is still unclear and lacking. Therefore, this study analyzed the effects of cinnamaldehyde, vitamin C, and their combination on the growth, carcass, and intestinal health of broilers.

## 2. Results

### 2.1. Effects of Dietary CA + VC on Growth and Slaughter Performance of Broilers

Feeding CA and VC had no significant interaction effect on growth performance criteria after 21 and 42 days in broilers (*p* > 0.05). Adding vitamin C (VC^+^) exhibited a trend towards increasing BWG (*p* = 0.094) in the early phase (1–21 d) compared to not adding vitamin C (VC^−^). At the same time, the inclusion of CA had no impact on broiler performance (Table 1).

Feeding CA and VC had no significant interaction effect on slaughter performance criteria after 21 and 42 days in broilers (*p* > 0.05). VC^+^ to the diet significantly increased breast muscle rate in 21-day-old broilers (5.33%, *p* < 0.05), reduced drip loss in 42-day-old broilers (20.68%, *p* < 0.05), and showed a trend towards increased breast muscle rate in 42-day-old broilers (7.09%, *p* = 0.097) compared to VC^−^. However, VC^+^ significantly reduced the abdominal fat rate for 42-day-old broilers (23.43%, *p* < 0.05) as compared to VC^−^. Adding CA alone had no effect on the slaughter performances of broilers (Table 2).

### 2.2. Effects of Dietary CA + VC on Duodenal Morphology of Broilers

Feeding CA and VC had no significant interaction effect on duodenal morphology criteria after 21 and 42 days in broilers (*p* > 0.05). The inclusion of cinnamaldehyde (CA^+^) in the diet significantly increased the CD (14.44%, *p* < 0.01) and reduced the VH/CD (21.25%, *p* < 0.01) at 42 d as compared to no added cinnamaldehyde (CA^−^). VC^+^ showed a trend towards increasing duodenal VH (9.94%, *p* = 0.088) at 21 d, decreased CD (12.61%, *p* < 0.05), and increased VH/CD (19.94%, *p* < 0.05) at 42 d as compared to VC^−^ (Table 3).

### 2.3. Effects of Dietary CA + VC on Jejunum Antioxidant Capacity of Broilers

Feeding CA and VC exhibited a significant interaction effect on broiler jejunal GSH-PX activity (*p* < 0.01), T-AOC, and MDA (*p* < 0.05) after 21 days. The inclusion of CA significantly decreased GSH-PX activity and increased MDA content (*p* < 0.05). The inclusion of VC significantly decreased GSH-PX activity and T-AOC (*p* < 0.05). When compared with CA, the inclusion of VC and CA + VC significantly decreased MDA content (*p* < 0.05) (Table 4).

Feeding CA and VC exhibited a significant interaction effect on broiler jejunal T-AOC (*p* < 0.05) after 42 days. The inclusion of CA, VC, and CA + VC significantly decreased T-AOC (*p* < 0.05). CA^+^ in the diet significantly decreased the SOD activity (*p* < 0.05) and the GSH content (*p* < 0.01) at 42 d as compared to CA^−^. VC^+^ in the diet significantly increased the GSH content (*p* < 0.01) and showed a trend towards increasing the MDA content (*p* = 0.090) at 42 d as compared to VC^−^ (Table 4). 

### 2.4. Effects of Dietary CA + VC on Intestinal mRNA Expression of Broilers

#### 2.4.1. Expression of Jejunal Barrier Gene mRNA

The supplementation of CA and VC in the diet generated an interactive effect on the mRNA expression of *Claudin-1* in the jejunum (*p* < 0.05) at 21 d. The addition of VC significantly upregulates the mRNA expression of *Claudin-1* (*p* < 0.05). VC^+^ in the diet significantly decreased the mRNA expression of *Occludin* (*p* < 0.05) and increased the mRNA expression of Zonula occludens-1 (*ZO-1*) (*p* < 0.01) at 21 d as compared to VC^−^ (Table 5).

The supplementation of CA and VC in the diet interactively affected the mRNA expression of *Occludin*, *Claudin-1*, *ZO-1*, and *Mucin-2* in the jejunum (*p* < 0.05) at 42-d. Adding CA + VC significantly boosted the mRNA expression of *Occludin*, *Claudin-1*, and *Mucin-2* (*p* < 0.01). The inclusion of CA significantly increased the mRNA expression of *Occludin*, *Claudin-1*, and *Mucin-2* (*p* < 0.05). The inclusion of VC significantly increased the mRNA expression of *Occludin*, *Claudin-1*, and *ZO-1* (*p* < 0.05). Compared to CA alone, adding CA + VC significantly reduced *Occludin* mRNA expression and increased the mRNA expression of *Claudin-1* and *Mucin-2* (*p* < 0.01). The inclusion of CA + VC significantly decreased the mRNA expression of *Claudin-1* and *ZO-1* and increased the mRNA expression of *Occludin* and *Mucin-2*, as compared to VC alone (*p* < 0.01). When compared with CA solely, the inclusion of VC significantly down-regulates the mRNA expression of *Occludin* and *Mucin-2* while upregulating the mRNA expression of *Claudin-1* and *ZO-1* (*p* < 0.01) (Table 5).

#### 2.4.2. Expression of Jejunal Antioxidant Gene mRNA

There was no interaction observed in the antioxidant gene mRNA expression in the jejunum of 21-day-old broilers between CA and VC (*p* > 0.05). CA^+^ in the diet led to a significant increase in the mRNA expression of *CAT* and superoxide dismutase 1 (*SOD1*) for 21 d broilers (*p* < 0.01) as compared to CA^−^. VC^+^ in the diet led to a significant increase in the mRNA expression of Glutathione Peroxidase 1 (*GPX-1*), *CAT*, and *SOD1* for 21 d broilers (*p* < 0.01) as compared to VC^−^ (Table 6).

There was a significant interaction for the mRNA expression of *CAT* (*p* < 0.05) and *SOD1* (*p* < 0.01) but had no interactive effect on the mRNA expression of *GPX-1* in the jejunum of 42-day-old broilers when CA and VC were added to the diet. The addition of CA + VC significantly increased *CAT* mRNA expression (*p* < 0.01) and decreased *SOD1* mRNA expression (*p* < 0.01). The addition of CA significantly increased *CAT* mRNA expression (*p* < 0.01) and decreased *SOD1* mRNA expression (*p* < 0.01). The inclusion of VC significantly decreased the mRNA expression of *SOD1* (*p* < 0.01). The addition of CA + VC significantly decreased *CAT* mRNA expression compared to CA alone (*p* < 0.01). The mRNA expression of *CAT* was significantly increased in the CA + VC (*p* < 0.01) compared with VC alone. When compared with CA, the inclusion of VC significantly decreased the mRNA expression of *CAT* and *SOD1* (*p* < 0.01). CA^+^ in the diet significantly increased the mRNA expression of *GPX-1* (*p* < 0.01) at 42 d as compared to CA^−^. VC^+^ in the diet significantly decreased the mRNA expression of *GPX-1* (*p* < 0.01) at 42 d as compared to VC^−^ (Table 6).

#### 2.4.3. Expression of Jejunal Inflammation-Related and Apoptosis-Related mRNA

A significant interactive effect was observed on the mRNA expression of interleukin 10 (*IL-10*), interleukin 6 (*IL-6*), tumor necrosis factor-alpha (*TNF-α*), and myeloid differentiation factor 88 (*MyD-88*) in the jejunum of 21-day-old broilers when CA and VC were incorporated into the diet (*p* < 0.01). The addition of CA + VC significantly reduced the mRNA expression of *IL-10*, *IL-6*, and tumor necrosis factor-alpha (*TNF-α*) (*p* < 0.01) and concurrently increased the mRNA expression of *MyD-88* (*p* < 0.01). The inclusion of CA significantly decreased the mRNA expression of *IL-10*, *IL-6*, and *TNF-α* (*p* < 0.01) while significantly increasing the expression of *MyD-88* (*p* < 0.01). The addition of VC significantly decreased the mRNA expression of *IL-10*, *IL-6*, and *TNF-α* (*p* < 0.01) while increasing the mRNA expression of *MyD-88* (*p* < 0.01). When compared with CA alone, the inclusion of CA + VC significantly decreased the mRNA expression of *IL-10* and increased the mRNA expression of *MyD-88* (*p* < 0.01). When compared with VC individually, the inclusion of CA + VC significantly decreased the mRNA expression of *IL-10* and *TNF-α* while increasing the mRNA expression of *MyD-88* (*p* < 0.01). The inclusion of VC significantly decreased the mRNA expression of *MyD-88* and increased the mRNA expression of *TNF-α* (*p* < 0.01) as compared with CA.CA^+^ in the diet significantly decreased the mRNA expression of interleukin-1 beta (*IL-1β*) and significantly increased the mRNA expression of nuclear factor-kappa B (*NF-κB*) (*p* < 0.01) at 21-d as compared to CA^−^. VC^+^ in the diet significantly increased the mRNA expression of *NF-κB* while decreasing the mRNA expression of toll-like receptor 2 (*TLR-2*) (*p* < 0.01) and showed a decreasing trend for the mRNA expression of toll-like receptor 4 (*TLR-4*) (*p* = 0.089) at 21 d as compared to VC^−^ (Table 7).

A significant interactive effect was observed for the mRNA expression of *IL-10*, *IL-6*, *TNF-α*, *IL-1β*, *TLR-4* (*p* < 0.01), and *NF-κB* (*p* < 0.05) in the jejunum of 42-day-old broilers when CA and VC were included in the diet. The inclusion of CA + VC significantly decreased the mRNA expression of *IL-10*, *IL-6*, *TNF-α*, and *IL-1β* (*p* < 0.01) while increasing the mRNA expression of *NF-κB* and *TLR-4* (*p* < 0.01). The inclusion of CA significantly decreased the mRNA expression of *IL-10*, *IL-6*, *TNF-α*, *IL-1β*, and *TLR-4* (*p* < 0.01) and increased the mRNA expression of *NF-κB* (*p* < 0.01). Adding VC significantly reduced the mRNA expression of *IL-10*, *IL-6*, *TNF-α*, and *IL-1β* (*p* < 0.01) while significantly increasing the mRNA expression of *NF-κB* (*p* < 0.05). The inclusion of CA + VC significantly increased the mRNA expression of *IL-1β*, *NF-κB*, and *TLR-4* (*p* < 0.01) as compared with CA separately. When compared with VC, the inclusion of CA + VC significantly decreased the mRNA expression of *IL-6* and increased the mRNA expression of *IL-1β*, *NF-κB*, and *TLR-4* (*p* < 0.01). When compared with CA, the inclusion of VC significantly decreased the mRNA expression of *NF-κB* while increasing the mRNA expression of *IL-6* and *TLR-4* (*p* < 0.01). CA^+^ in the diet significantly decreased the mRNA expression of *TLR-2* (*p* < 0.05) and increased the mRNA expression of *MyD-88* (*p* < 0.01) at 42-d as compared to CA^−^. VC^+^ in the diet significantly increased the mRNA expression of *MyD-88* and *TLR-2* at 42-d as compared to VC^−^ (*p* < 0.01) (Table 7).

## 3. Discussion

Recently, adding plant extracts and vitamins to the diet for better broiler production and health has become more popular [12,15]. In this study, the effects of cinnamaldehyde, vitamin C, and their combination on the growth performances, carcass attributes, and intestinal health of broilers were evaluated. This study showed that CA, VC, or a combination of CA + VC to broiler feed had no significant interaction effect on the growth performances of broilers. In contrast to our results, including various amounts (basic diet plus 0.25%, 0.50%, 0.75%, 1.0%) of cinnamon powder increased the BW of broilers [16]. Similar to our results, cinnamon powder does not affect the growth of broilers [17]. However, adding VC to the diet in the rearing phase increased BWG. In similarity with our results, Lopes et al. [13] and Khan et al. [18] found that supplementing vitamin C in the early stages of the broiler led to better WG and FCR. However, Jahejo et al. [19] reported that adding VC to broiler feed had no significant effect on productivity. The CA enhances productivity by acting as an antibacterial and anti-inflammatory agent in the intestine, reducing the pathogen numbers. Moreover, the VC can act as an anti-stress agent. Therefore, differences in various study results might be due to the degree of infection or stress that showed variation in the effects of additives.

In this study, adding VC could boost the breast muscle rate of broilers and reduce the abdominal fat rate. The previous studies showed a reduction in abdominal fat with added VC [20], which supports our findings. The fat content is important for water retention and oxidation processes in stored meat, affecting water-holding capacity [21,22]. This study showed that dietary VC reduced drip loss by potentially decreasing abdominal fat in broilers and delaying protein and lipid oxidation in chicken meat.

The intestinal morphology is important for broiler growth and health. A higher intestinal VH increases nutrient absorption, benefiting broiler growth [23]. The higher the VH/CD, the more beneficial it is for the absorption of nutrients in broilers [24]. In this study, the addition of CA in the diet increased the CD and reduced the VH/CD in broilers at 42-d. The addition of VC in the diet increased the VH (21 d) and VH/CD at (42 d) and reduced the CD (42 d) in the duodenum. Excessive amounts of CA can lead to adverse effects on morphology [10,25]; however, the CA + VC could lessen these effects, as shown in this study. The CA can stimulate intestinal tissues by inhibiting pathogenic microorganisms, which might reduce the surface area of the intestine.

Tight junctions (TJ) in the intestine help in nutrient absorption and immunity and control its barrier function. The ZO-1 forms tight junctions by connecting proteins like Occludin and Claudin, regulating diverse signaling pathways [26]. The Claudins interact with the morphology and function of tight junctions. Damage to the Claudins disrupts the intestinal barrier function and enhances mucosal permeability [27]. Occludin is crucial for ensuring the structural integrity of the intestinal mucosa in broilers and preventing it from harmful substances [28]. Mucin-2 helps in resisting the invasion of pathogens and protecting intestinal tissues [29]. This study found that the addition of VC had a beneficial effect on the intestinal barrier mRNA expression. The CA can counteract the adverse effects of VC on the expression of *Occludin* at 42 d, and the CA + VC can increase the expression of *Mucin-2*. Adding VC and CA improves the intestinal barrier, preserves the epithelial layer, and reduces intestinal permeability in broilers. However, the effects of CA and VC are dosage-dependent, and excessive amounts may have adverse effects [10].

The intestinal epithelium is exposed to luminal oxidants and is vulnerable to oxidative damage [30]. The antioxidant defense system, comprising enzymatic and non-enzymatic antioxidants, combats oxidative damage. Enzymatic antioxidants GPX, CAT, and SOD combat harmful superoxide free radicals, contributing to antioxidant and anti-aging effects. The mechanism of action is that SOD converts superoxide radicals to H_2_O_2_ and O_2_, followed by GPX and CAT independently converting H_2_O_2_ into H_2_O and O_2_ [31]. In this study, the VC upregulated the *GPX-1*, *CAT*, and *SOD1* mRNA expression of 21-d and downregulated the *GPX-1* and *SOD1* of 42-day-old broilers. The addition of CA upregulated the *CAT* (21 d and 42 d) and *GPX-1* (42-d) mRNA expression. Gan et al. [32] reported that the requirement of VC in broilers increased as they grew. This is attributed to physiological changes affecting their ability to synthesize vitamin C. L-gluconolactone oxidase (GLO) is crucial for the final step of vitamin C synthesis in animal tissues. In broilers, GLO enzyme activity increased after birth but declined as they aged from 1 to 42 days [32]. In later growth stages, broilers may not synthesize enough VC to meet their needs [33], potentially causing variations in VC effects on mRNA expression levels of antioxidants at different ages. Gan et al. found that adding 0.25 g VC/kg to the diet of old laying hens increased liver T-AOC and total GSH; however, excessive supplementation (above 1 g/kg) could inhibit the secretion of endogenous antioxidants like T-AOC and GSH, which supports our results [34]. In this study, the combination of CA + VC upregulated antioxidant gene mRNA expression; however, it did not significantly affect enzymatic activity levels. GSH, a non-enzymatic antioxidant, scavenges free radicals, decomposes hydrogen peroxide via the GSH-PX enzyme, and is a vital measure of the body’s antioxidant capacity. This study found that CA reduced the GSH (42 d) content. Similar to our results, cinnamaldehyde effectively depleted GSH, consequently increasing the generation of ROS in prostate cancer-associated fibroblasts [35]. Cinnamaldehyde potentially reacts with intracellular GSH by Michael Addition, lowering GSH levels [36], especially in older people. This suggests that excessively high doses of cinnamaldehyde may harm intestinal antioxidant capacity. The MDA is a useful biomarker of oxidative stress in the body. In this study, CA increased the MDA (21-d) content in the jejunum; however, CA + VC could counteract this effect. The T-AOC measures the overall antioxidant level, providing a significant and comprehensive evaluation of antioxidant capacity [37]. In this study, CA + VC reduced the T-AOC (42-d) level. Excessive VC supplementation might inhibit endogenous antioxidant enzymes [34], while cinnamaldehyde could reduce intracellular GSH levels [36]. Therefore, the effect of VC [38] and CA [30] on intestinal antioxidant capacity is dose-dependent and varies with the age of broilers, as shown in this study.

The Toll-like receptors (TLRs) have pattern recognition receptors identifying pathogen-associated molecular patterns (PAMPs) and transmit downstream signals upon ligands-and-receptor binding [39]. The TLR2 and TLR4 can recognize PAMPs such as lipopolysaccharides [40]. After ligand-receptor binding, the adaptor protein Myd88 is recruited, activating NF-κB and producing pro-inflammatory cytokines (IL-6 and IL-1β) to regulate immune responses [41]. NF-κB, a vital transcription factor in the TLR signaling pathway, activates and enters the nucleus to promote the expression of target genes [42]. The addition of CA and VC in this study upregulated the *TLR-4* (42-d) in broilers. The CA, VC, and CA + VC upregulated the expression of *NF-κB* and *MyD-88*. Maintaining or increasing *TLR-4* and *TLR-2* mRNA expression implies a responsive immune system to potential pathogens, while a decrease may impair the immune system’s ability to recognize and respond to pathogens [43]. Higher mRNA levels of NF-κB and MyD-88 suggest an intensified immune response [41]. The cytokines play a crucial role in regulating the inflammatory response [44]. The IL-10 is a potential anti-inflammatory cytokine secreted in the late stage of inflammation and has pro-inflammatory factors [45]. The IL-6 binds to its receptor, triggers cell differentiation, and produces inflammatory mediators like IL-1, IL-6, and IL-8. Additionally, IL-6 stimulates macrophage and lymphocyte proliferation, promoting immune responses [46]. TNF-α, a crucial cytokine, stimulates cell proliferation or signals apoptosis, influencing the synthesis and release of pro-inflammatory effects [47]. IL-1β, a key pro-inflammatory mediator, boosts the activity of vascular endothelial-leukocyte adhesion molecules, attracting inflammatory cells to the site of intestinal changes in animals and triggering an inflammatory response [48]. In this study, CA down-regulates *IL-1β* and *TNF-α*. Chun et al. reported that trans-cinnamic aldehyde inhibited inflammatory responses and bacterial survival through autophagy activation [49], which aligns with our findings. It suggests that combining VC and CA can help maintain immune function in broilers.

## 4. Materials and Methods

### 4.1. Ethics Statement

The animal procedures adhered to the Laboratory Animal Regulations of Beijing and were approved by the Laboratory Animal Ethical Committee at China Agricultural University (approval number: AW04129102-1-1).

### 4.2. Birds, Diets, and Experimental Design

A total of 288 one-day-old female Arbor Acres broilers (obtained from Beijing Poultry Breeding Co., Ltd., Beijing, China) were randomly distributed into four dietary treatments, with six replicate cages and 12 birds each. The control group (CON) was fed a basal diet. The treatment diets were supplemented with cinnamaldehyde (CA) 300 g/t, vitamin C (VC) 300 g/t, and a combination of cinnamaldehyde 300 g/t and vitamin C 300 g/t (CA + VC), respectively.

The basal diet was formulated to meet or exceed the nutrient requirement for broilers recommended by NRC (1994). The experiment was performed at the Zhuozhou breeding base of China Agricultural University and lasted for 42 d. The starter phase was from 1 to 21 d, the finisher phase was from 22 to 42 d, and the whole phase was from 1 to 42 d. The ingredient and nutrient composition of the basal diets for the starter (0–21 d) and finisher (22–42 d) phases are shown in Table 8. The starter diet was pelleted and crumbled, while the finisher’s diet was fed in pelleted form. The basal diet and treatment diets were all fed from 1 d to 42 d. Water was provided ad libitum using a nipple-type drinker. The birds were immunized against NDV at the age of 7 days. The room temperature was maintained at 33–35 °C during the first 3 days, which was gradually decreased to 25 °C and 22–25 °C for the remainder of the trial. A standard lighting regime was followed: 23 h of light and 1 h of darkness for the first 8 days, followed by 20 h of light and 4 h of darkness from 9 d until the end of the trial. 

### 4.3. Performance Parameters

At 21 and 42 d, feed withdrawal was carried out 12 h before obtaining samples. The individual weights and feed intake were recorded for each cage to calculate the body weight gain (BWG), feed intake (FI), and feed-to-gain ratio (F/G). Furthermore, daily mortality records for the broilers were kept, allowing the determination of survival rates for each group.

The BWG, FI, F/G, and survival rates were calculated with the following formulas:

BWG = (final chicken weight (kg)/final chicken number) − (initial chicken weight (kg)/initial chicken number)

F/G = total feed intake (kg)/(final chicken weight (kg) − initial chicken weight (kg))

Dead chicken feed intake(kg) = (dead chicken bodyweight (g) − initial chicken weight (g))/1000 × FI

FI = (total feed intake (kg) − dead chicken feed intake (kg))/number of live chickens

Survival ratios (%) = final chicken number/initial chicken number

### 4.4. Sample Collection and Parameters Determination

At 21 and 42 d, one chicken per replicate was randomly selected, weighed, stunned, and bled. Three pieces of jejunum (0.5 cm) samples were collected and cleaned with sterile PBS, snap frozen in liquid nitrogen, and then transferred to −80 °C for cryopreservation to extract RNA, then detect the mRNA expression of the intestinal barrier, inflammation factors, and antioxidant genes by the method described in Section 4.5.3; the jejunal mucosa was scraped and snap frozen in liquid nitrogen, transferred to −80 °C and frozen for antioxidant indexes. Samples of the middle part of the duodenum (1 cm) were taken and fixed in 4% paraformaldehyde for morphological analysis.

At 21 and 42 d, one chicken per replicate was randomly selected. After being weighed (live weight before slaughter), stunned, and bled, the chickens were treated with hot water to remove their feathers and then weighed to obtain the carcass weight and calculate the dressing percentage. After removing the trachea, esophagus, crop, intestine, spleen, pancreas, heart, liver, glandular stomach, muscular stomach, and abdominal fat, the chickens were weighted to obtain the total evisceration ratio. At 21 d, the breast muscles on both sides were removed to obtain the ratio of breast muscle. At 42 d, the breast muscles on both sides and the abdominal fat (including all the fat around the abdomen and the muscular stomach) were also removed to calculate the ratios of breast muscle and abdominal fat. At 42 d, a 10 g long piece of meat from the left breast muscle was hung and placed at 4 °C for 24 h, and the drip loss was calculated.

The breast muscle percentage, dressing percentage, total evisceration ratio, abdominal fat percentage, and drip loss were calculated with the following formulas:

Breast muscle ratio (%) = weight of breast muscle (g) × 100/carcass weight (g)

Dressing ratio (%) = carcass weight (g) × 100/live weight before slaughter (g)

Total evisceration ratio (%) = total eviscerated weight (g) × 100/live weight before slaughter (g)

Abdominal fat ratio (%) = weight of abdominal fat (g) × 100/carcass weight (g)

Drip loss (%) = (weight of meat sample before hanging (g) − weight of meat sample after 24 h hanging (g)) × 100/weight of meat sample before hanging (g)

### 4.5. Sampling for RNA and Antioxidant Indices

#### 4.5.1. Intestinal Histomorphology

The duodenum was fixed for 24 h, dehydrated, embedded in paraffin, sectioned, and then stained with hematoxylin-eosin (HE) and Periodic Acid-Schiff stain (PAS) to show the structure of intestinal villi. The villus height (VH) was based on the vertical height from the opening of the intestinal gland to the top of the villi, and the crypt depth (CD) was based on the vertical height from the muscularis mucosa to the opening of the intestinal gland.

#### 4.5.2. Intestinal Antioxidant Function

The jejunal mucosa was homogenized in 0.9% precooled saline and centrifuged at 3000 rpm for 10 min at 4 °C. The supernatant was stored at −4 °C for further analysis. The assays for glutathione peroxidase (GSH-PX, cat# A005-1), total antioxidant capacity (T-AOC, cat# A015-2-1), superoxide dismutase (SOD, cat# A001-3), reduced glutathione (GSH, cat# A006-2-1), catalase (CAT, cat# A007-1-1), and malondialdehyde (MDA, cat# A003-1) were conducted using the Nanjing Institute of Bioengineering reagent kits, following the manufacturer’s instructions.

#### 4.5.3. Relative mRNA Expression in the Intestine

The RNA was extracted from the jejunal samples using a Trizol reagent (TaKaRa, Osaka, Japan). The synthesis of cDNA was carried out according to the instructions provided in the Prime-Script-TM RT reagent Kit with gDNA Eraser (Perfect Real Time) kit (TaKaRa, Osaka, Japan). The primer sequences are provided in Table 9, and all primers used in this study were synthesized by Sangon Biotech (Shanghai) Co., Ltd. (Shanghai, China). For relative mRNA expression analysis of the target genes, real-time fluorescence quantitative PCR (RT-qPCR) was used. The procedure followed the instructions of the TB Green^®^ Premix Ex TaqTM (Tli RNaseH Plus) kit (RR420A, TaKaRa, Osaka, Japan). The reaction mixture consisted of 5 μL TB Green Premix Ex Taq II, 0.4 μL PCR Forward Primer, 0.4 μL PCR Reverse Primer, 0.2 μL ROX Reference Dye II, 2 μL cDNA, and 2 μL RNase Free H_2_O, totaling 10 μL. The β-actin gene was employed as the reference gene to determine the Ct values of the target genes. The 2^−ΔΔCt^ method was applied for calculations, analyzing the relative mRNA expression levels of the target genes.

### 4.6. Statistical Analysis

Data were analyzed using a complete randomized design to check for homogeneity of variances, employing SPSS-26 software (version 26.0, SPSS Institute, Chicago, IL, USA). A two-way analysis of variance (two-way ANOVA) was conducted to determine the presence of interactions between treatment groups. The Least significant difference (LSD) was performed using Duncan`s multiple comparison tests. The main effects were compared in the case of no-significant interaction effects. The statistical significance was defined as *p* < 0.05, indicating significant differences. A range of 0.05 < *p* < 0.10 suggested a notable trend toward differences.

## 5. Conclusions

In this study, dietary supplementation of cinnamaldehyde did not affect the growth performance or slaughter performance of broilers but improved barrier function and reduced the expression of inflammatory factors. Vitamin C, on the other hand, enhanced early broiler growth and slaughter performance, intestinal health, and barrier function and helped maintain a balanced immune response. Combining CA + VC benefits broiler carcass quality and intestinal health.

## Figures and Tables

**Table 1:** Effects of dietary cinnamaldehyde and vitamin C on growth performance of broilers.

Item	BWG ^1^/kg	FI ^2^/kg	F/G ^3^	Survival Rate/%
	1–21 d			
CON	0.71 ± 0.05	1.05 ± 0.07	1.47 ± 0.05	100.00 ± 0.00
CA	0.74 ± 0.03	1.07 ± 0.05	1.45 ± 0.03	100.00 ± 0.00
VC	0.76 ± 0.04	1.09 ± 0.05	1.43 ± 0.02	100.00 ± 0.00
CA + VC	0.75 ± 0.04	1.09 ± 0.05	1.45 ± 0.03	100.00 ± 0.00
cinnamaldehyde				
-	0.74 ± 0.05	1.07 ± 0.06	1.45 ± 0.04	100.00 ± 0.00
+	0.75 ± 0.04	1.08 ± 0.05	1.45 ± 0.03	100.00 ± 0.00
vitamin C				
-	0.73 ± 0.04	1.06 ± 0.06	1.46 ± 0.04	100.00 ± 0.00
+	0.76 ± 0.04	1.09 ± 0.05	1.44 ± 0.03	100.00 ± 0.00
*p*-value				
cinnamaldehyde	0.566	0.615	0.825	-
vitamin C	0.094	0.228	0.231	-
interaction	0.259	0.630	0.125	-
	22–42 d			
CON	1.55 ± 0.17	2.85 ± 0.20	1.85 ± 0.10	98.33 ± 4.08
CA	1.51 ± 0.17	2.78 ± 0.21	1.85 ± 0.20	98.49 ± 3.71
VC	1.53 ± 0.18	2.72 ± 0.14	1.80 ± 0.16	98.49 ± 3.71
CA + VC	1.57 ± 0.17	2.75 ± 0.12	1.77 ± 0.14	100.00 ± 0.00
cinnamaldehyde				
-	1.54 ± 0.17	2.78 ± 0.18	1.82 ± 0.13	98.41 ± 3.72
+	1.54 ± 0.16	2.76 ± 0.17	1.81 ± 0.17	99.24 ± 2.62
vitamin C				
-	1.53 ± 0.16	2.81 ± 0.20	1.85 ± 0.15	98.41 ± 3.72
+	1.55 ± 0.17	2.74 ± 0.13	1.78 ± 0.14	99.24 ± 2.62
*p*-value				
cinnamaldehyde	0.983	0.766	0.842	0.558
vitamin C	0.789	0.311	0.300	0.558
interaction	0.578	0.492	0.763	0.595
	1–42 d			
CON	2.26 ± 0.22	3.89 ± 0.26	1.73 ± 0.08	98.61 ± 3.40
CA	2.25 ± 0.19	3.85 ± 0.24	1.72 ± 0.13	98.61 ± 3.40
VC	2.29 ± 0.21	3.81 ± 0.19	1.67 ± 0.09	98.61 ± 3.40
CA + VC	2.31 ± 0.21	3.84 ± 0.17	1.66 ± 0.09	100.00 ± 0.00
cinnamaldehyde				
-	2.27 ± 0.21	3.85 ± 0.22	1.70 ± 0.09	98.61 ± 3.24
+	2.29 ± 0.19	3.84 ± 0.20	1.69 ± 0.11	99.31 ± 2.40
vitamin C				
-	2.26 ± 0.19	3.87 ± 0.24	1.72 ± 0.10	98.61 ± 3.24
+	2.30 ± 0.20	3.83 ± 0.17	1.67 ± 0.09	99.31 ± 2.40
*p*-value				
cinnamaldehyde	0.897	0.918	0.778	0.570
vitamin C	0.572	0.630	0.199	0.570
interaction	0.809	0.678	0.981	0.570

Abbreviations: CON, basal diet; CA, basal diet with 300 g/t of cinnamaldehyde; VC, basal diet with 300 g/t of vitamin C; CA + VC, basal diet with 300 g/t of cinnamaldehyde and 300 g/t of vitamin C in combination. The main effects were compared in the case of no-significant interaction effects. “+” represent to add, “-” represents no added. ^1^ BWG: body weight gain, kg/bird. ^2^ FI: feed intake, kg/bird. ^3^ F/G: feed-to-weight ratio.

**Table 2:** Effects of dietary cinnamaldehyde and vitamin C on slaughter performance of broilers.

Item	21 d	42 d				
	BMR ^1^/%	BMR/%	DR ^2^/%	TER ^3^/%	AFR ^4^/%	DL ^5^/%
CON	18.01 ± 1.55	27.30 ± 4.74	93.86 ± 1.11	75.59 ± 1.75	2.33 ± 0.84	3.19 ± 0.81
CA	17.97 ± 0.16	28.31 ± 2.08	91.27 ± 7.35	75.01 ± 1.56	2.46 ± 0.68	3.28 ± 0.79
VC	18.85 ± 0.35	29.37 ± 2.01	94.18 ± 0.68	74.81 ± 1.98	1.71 ± 0.25	2.20 ± 0.57
CA + VC	19.05 ± 1.55	30.17 ± 1.25	94.63 ± 0.41	75.67 ± 1.48	1.96 ± 0.50	2.93 ± 0.47
cinnamaldehyde						
-	18.43 ± 1.16	28.34 ± 3.64	94.02 ± 0.90	75.20 ± 1.83	2.02 ± 0.67	2.70 ± 0.84
+	18.51 ± 1.19	29.24 ± 1.90	92.95 ± 5.26	75.34 ± 1.49	2.21 ± 0.63	3.11 ± 0.65
vitamin C						
-	17.99 ± 1.05 ^b^	27.80 ± 3.53	92.56 ± 5.19	75.30 ± 1.61	2.39 ± 0.73 ^a^	3.24 ± 0.76 ^a^
+	18.95 ± 1.08 ^a^	29.77 ± 1.65	94.40 ± 0.59	75.24 ± 1.73	1.83 ± 0.40 ^b^	2.57 ± 0.63 ^b^
*p*-value						
cinnamaldehyde	0.863	0.453	0.571	0.855	0.450	0.152
vitamin C	0.047	0.097	0.218	0.939	0.036	0.025
interaction	0.792	0.935	0.333	0.313	0.817	0.261

Abbreviations: CON, basal diet; CA, basal diet with 300 g/t of cinnamaldehyde; VC, basal diet with 300 g/t of vitamin C; CA + VC, basal diet with 300 g/t of cinnamaldehyde and 300 g/t of vitamin C in combination. Different letters in the same column indicate significant differences (*p* < 0.05), and the same letter means no significant difference (*p* > 0.05). The main effects were compared in the case of no-significant interaction effects. “+” represent to add, “-” represents no added. ^1^ BMR: breast muscle rate, %. ^2^ DR: dressing rate, %. ^3^ TER: total evisceration rate, %. ^4^ AFR: abdominal fat rate, %. ^5^ DL: drip loss, %.

**Table 3:** Effects of dietary cinnamaldehyde and vitamin C on duodenal morphology of broilers.

Items	21d			42 d		
	VH ^1^ (μm)	CD ^2^ (μm)	VH/CD	VH (μm)	CD (μm)	VH/CD
CON	18.01 ± 1.55	27.30 ± 4.74	93.86 ± 1.11	75.59 ± 1.75	2.33 ± 0.84	3.19 ± 0.81
CA	17.97 ± 0.16	28.31 ± 2.08	91.27 ± 7.35	75.01 ± 1.56	2.46 ± 0.68	3.28 ± 0.79
VC	18.85 ± 0.35	29.37 ± 2.01	94.18 ± 0.68	74.81 ± 1.98	1.71 ± 0.25	2.20 ± 0.57
CA + VC	19.05 ± 1.55	30.17 ± 1.25	94.63 ± 0.41	75.67 ± 1.48	1.96 ± 0.50	2.93 ± 0.47
cinnamaldehyde						
-	1341.01 ± 185.44	201.77 ± 22.35	6.74 ± 1.23	1394.00 ± 218.97	203.26 ± 31.00 ^b^	6.97 ± 1.26 ^a^
+	1363.42 ± 190.73	204.77 ± 32.57	6.80 ± 1.37	1346.97 ± 203.80	244.01 ± 35.15 ^a^	5.62 ± 1.12 ^b^
vitamin C						
-	1288.08 ± 161.39	195.25 ± 16.65	6.66 ± 1.12	1324.92 ± 179.19	238.63 ± 42.35 ^a^	5.72 ± 1.25 ^b^
+	1416.36 ± 189.78	211.28 ± 33.88	6.88 ± 1.46	1416.05 ± 232.49	208.64 ± 28.59 ^b^	6.86 ± 1.25 ^a^
*p*-value						
cinnamaldehyde	0.757	0.795	0.917	0.600	0.004	0.006
vitamin C	0.088	0.175	0.686	0.314	0.025	0.017
interaction	0.158	0.941	0.419	0.955	0.561	0.987

Abbreviations: CON, basal diet; CA, basal diet with 300 g/t of cinnamaldehyde; VC, basal diet with 300 g/t of vitamin C; CA + VC, basal diet with 300 g/t of cinnamaldehyde and 300 g/t of vitamin C in combination. Different letters in the same column indicate significant differences (*p* < 0.05), and the same letter means no significant difference (*p* > 0.05). The main effects were compared in the case of no-significant interaction effects. “+” represent to add, “-” represents no added. ^1^ VH: villus height, μm. ^2^ CD: crypt depth, μm.

**Table 4:** Effects of dietary cinnamaldehyde and vitamin C on jejunum antioxidant capacity of broilers.

Items	GSH-PX ^1^ (Active Unit)	T-AOC ^2^ (mmol/g)	SOD ^3^ (U/mgport)	GSH ^4^ (μmol/gprot)	CAT ^5^ (U/mgprot)	MDA ^6^ (nmol/mgprot)
	21 d					
CON	58.15 ± 27.24 ^a^	0.21 ± 0.03 ^a^	24.71 ± 4.08	66.69 ± 16.84	0.05 ± 0.06	0.18 ± 0.05 ^b^
CA	31.31 ± 1.73 ^b^	0.19 ± 0.02 ^ab^	23.04 ± 3.76	57.75 ± 10.89	0.05 ± 0.05	0.27 ± 0.05 ^a^
VC	33.86 ± 3.96 ^b^	0.18 ± 0.03 ^b^	24.17 ± 2.27	55.96 ± 22.40	0.04 ± 0.03	0.20 ± 0.03 ^b^
CA + VC	48.29 ± 14.79 ^ab^	0.20 ± 0.02 ^ab^	24.51 ± 2.54	51.34 ± 5.70	0.06 ± 0.07	0.20 ± 0.04 ^b^
cinnamaldehyde						
-	46.00 ± 22.48	0.20 ± 0.03	24.44 ± 3.16	61.33 ± 19.71	0.05 ± 0.05	0.19 ± 0.04
+	39.80 ± 13.40	0.20 ± 0.02	23.78 ± 3.15	54.55 ± 8.94	0.05 ± 0.06	0.23 ± 0.06
vitamin C						
-	44.73 ± 23.14	0.20 ± 0.03	23.88 ± 3.84	62.22 ± 14.31	0.05 ± 0.05	0.22 ± 0.06
+	41.07 ± 12.78	0.19 ± 0.03	24.34 ± 2.30	53.65 ± 15.77	0.05 ± 0.06	0.20 ± 0.04
*p*-value						
cinnamaldehyde	0.343	0.920	0.622	0.291	0.700	0.033
vitamin C	0.574	0.713	0.731	0.185	0.838	0.176
interaction	0.004	0.049	0.460	0.733	0.675	0.016
	42 d					
CON	38.13 ± 9.77	0.21 ± 0.03 ^a^	31.07 ± 2.28	57.97 ± 5.50	0.06 ± 0.08	0.17 ± 0.02
CA	23.90 ± 14.41	0.16 ± 0.02 ^b^	26.74 ± 5.23	42.70 ± 5.28	0.06 ± 0.05	0.15 ± 0.04
VC	29.21 ± 8.17	0.17 ± 0.01 ^b^	29.00 ± 1.23	53.44 ± 5.97	0.11 ± 0.11	0.19 ± 0.02
CA + VC	34.47 ± 18.26	0.16 ± 0.01 ^b^	24.16 ± 8.40	52.18 ± 6.38	0.09 ± 0.09	0.17 ± 0.04
cinnamaldehyde						
-	33.67 ± 9.77	0.19 ± 0.03	30.03 ± 2.06 ^a^	55.71 ± 6.17 ^a^	0.09 ± 0.10	0.18 ± 0.02
+	29.18 ± 16.63	0.16 ± 0.02	25.45 ± 6.80 ^b^	47.44 ± 7.46 ^b^	0.07 ± 0.07	0.16 ± 0.04
vitamin C						
-	31.01 ± 13.89	0.19 ± 0.03	28.91 ± 4.46	50.33 ± 9.48 ^b^	0.06 ± 0.06	0.16 ± 0.03
+	31.84 ± 13.76	0.16 ± 0.01	26.58 ± 6.25	57.81 ± 8.32 ^a^	0.10 ± 0.10	0.18 ± 0.03
*p*-value						
cinnamaldehyde	0.417	0.001	0.040	<0.001	0.764	0.260
vitamin C	0.880	0.004	0.278	0.005	0.324	0.090
interaction	0.087	0.049	0.906	0.407	0.865	0.839

Abbreviations: CON, basal diet; CA, basal diet with 300 g/t of cinnamaldehyde; VC, basal diet with 300 g/t of vitamin C; CA + VC, basal diet with 300 g/t of cinnamaldehyde and 300 g/t of vitamin C in combination. Different letters in the same column indicate significant differences (*p* < 0.05), and the same letter means no significant difference (*p* > 0.05). The main effects were compared in the case of no-significant interaction effects. “+” represent to add, “-” represents no added. ^1^ GSH-PX: glutathione peroxidase, active unit. ^2^ T-AOC: total antioxidant capacity, mmol/g. ^3^ SOD: superoxide dismutase, U/mgport. ^4^ GSH: reduced glutathione, μmol/gprot. ^5^ CAT: catalase, U/mgprot. ^6^ MDA: malondialdehyde, nmol/mgprot.

**Table 5:** Effects of dietary cinnamaldehyde and vitamin C on jejunal barrier gene mRNA expression of broilers.

Items ^1^	21 d				42 d			
	Occludin	Claudin-1	ZO-1 ^1^	Mucin-2	Occludin	Claudin-1	ZO-1	Mucin-2
CON	1.00 ± 0.22	1.00 ± 0.25 ^b^	1.00 ± 0.16	1.00 ± 0.25	1.00 ± 0.31 ^c^	1.00 ± 0.37 ^d^	1.00 ± 0.08 ^b^	1.00 ± 0.20 ^c^
CA	0.93 ± 0.43	1.31 ± 0.29 ^ab^	1.51 ± 0.43	0.91 ± 0.21	4.57 ± 0.42 ^a^	6.41 ± 1.77 ^c^	1.21 ± 0.24 ^b^	2.83 ± 0.49 ^b^
VC	0.59 ± 0.36	1.58 ± 0.35 ^a^	1.75 ± 0.47	1.05 ± 0.75	0.23 ± 0.08 ^d^	14.98 ± 3.05 ^a^	1.57 ± 0.9 ^a^	1.40 ± 0.47 ^c^
CA + VC	0.79 ± 0.14	1.30 ± 0.35 ^ab^	2.01 ± 0.86	1.08 ± 0.12	1.89 ± 0.43 ^b^	11.48 ± 2.59 ^b^	1.04 ± 0.25 ^b^	4.96 ± 0.68 ^a^
cinnamaldehyde								
-	0.80 ± 0.35	1.29 ± 0.42	1.38 ± 0.52	1.02 ± 0.53	0.62 ± 0.46	7.99 ± 7.59	1.29 ± 0.31	1.20 ± 0.40
+	0.86 ± 031	1.30 ± 0.31	1.76 ± 0.70	0.99 ± 0.18	3.23 ± 1.46	8.94 ± 3.39	1.12 ± 0.25	3.89 ± 1.25
vitamin C								
-	0.97 ± 0.33 ^a^	1.15 ± 0.30	1.25 ± 0.41 ^b^	0.95 ± 0.22	2.78 ± 1.98	3.70 ± 3.08	1.11 ± 0.20	1.91 ± 1.02
+	0.69 ± 0.28 ^b^	1.44 ± 0.37	1.88 ± 0.67 ^a^	1.06 ± 0.51	1.06 ± 0.91	13.23 ± 3.26	1.30 ± 0.34	3.18 ± 1.94
*p*-value								
cinnamaldehyde	0.612	0.934	0.100	0.856	<0.001	0.299	0.043	<0.001
vitamin C	0.040	0.037	0.010	0.536	<0.001	<0.001	0.015	<0.001
interaction	0.315	0.033	0.575	0.721	<0.001	<0.001	<0.001	<0.001

Abbreviations: CON, basal diet; CA, basal diet with 300 g/t of cinnamaldehyde; VC, basal diet with 300 g/t of vitamin C; CA + VC, basal diet with 300 g/t of cinnamaldehyde and 300 g/t of vitamin C in combination. Different letters in the same column indicate significant differences (*p* < 0.05), and the same letter means no significant difference (*p* > 0.05). The main effects were compared in the case of no-significant interaction effects. “+” represent to add, “-” represents no added. ^1^ ZO-1: Zonula Occludens-1.

**Table 6:** Effects of dietary cinnamaldehyde and vitamin C on jejunal antioxidant gene mRNA expression of broilers.

Items	21 d			42 d		
	GPX-1 ^1^	CAT ^2^	SOD1 ^3^	GPX-1	CAT	SOD1
CON	1.00 ± 0.34	1.00 ± 0.21	1.00 ± 0.33	1.00 ± 0.38	1.00 ± 0.19 ^c^	1.00 ± 0.27 ^a^
CA	0.81 ± 0.19	2.23 ± 0.40	1.51 ± 0.23	1.62 ± 0.25	7.74 ± 0.91 ^a^	0.29 ± 0.07 ^b^
VC	1.29 ± 0.32	1.99 ± 0.82	1.52 ± 0.37	0.36 ± 0.03	1.66 ± 0.46 ^c^	0.04 ± 0.01 ^c^
CA + VC	1.20 ± 0.12	3.12 ± 0.89	2.76 ± 0.72	0.95 ± 0.09	6.32 ± 1.61 ^b^	0.17 ± 0.02 ^bc^
cinnamaldehyde						
-	1.15 ± 0.35	1.49 ± 0.77 ^b^	1.26 ± 0.43 ^b^	0.68 ± 0.42 ^b^	1.33 ± 0.48	0.52 ± 0.53
+	1.00 ± 0.25	2.67 ± 080 ^a^	2.13 ± 0.83 ^a^	1.28 ± 0.39 ^a^	7.03 ± 1.45	0.23 ± 0.08
vitamin C						
-	0.91 ± 0.28 ^b^	1.62 ± 0.71 ^b^	1.25 ± 0.38 ^b^	1.31 ± 0.44 ^a^	4.37 ± 3.58	0.64 ± 0.41
+	1.24 ± 0.23 ^a^	2.55 ± 1.01 ^a^	2.14 ± 0.85 ^a^	0.65 ± 0.31 ^b^	3.99 ± 2.69	0.10 ± 0.07
*p*-value						
cinnamaldehyde	0.186	<0.001	<0.001	<0.001	<0.001	<0.001
vitamin C	0.005	0.002	<0.001	<0.001	0.342	<0.001
interaction	0.624	0.842	0.057	0.896	0.015	<0.001

Abbreviations: CON, basal diet; CA, basal diet with 300 g/t of cinnamaldehyde; VC, basal diet with 300 g/t of vitamin C; CA + VC, basal diet with 300 g/t of cinnamaldehyde and 300 g/t of vitamin C in combination. Different letters in the same column indicate significant differences (*p* < 0.05), and the same letter means no significant difference (*p* > 0.05). The main effects were compared in the case of no-significant interaction effects. “+” represent to add, “-” represents no added. ^1^ GPX-1: glutathione peroxidase 1. ^2^ CAT: catalase. ^3^ SOD1: superoxide dismutase 1.

**Table 7:** Effects of dietary cinnamaldehyde and vitamin C on jejunal inflammation-related and apoptosis-related mRNA expression of broilers.

Items ^1^	IL-10 ^1^	IL-6 ^2^	TNF-α ^3^	IL-1β ^4^	NF-κB ^5^	MyD-88 ^6^	TLR-4 ^7^	TLR-2 ^8^
	21 d							
CON	1.00 ± 0.20 ^a^	1.00 ± 0.15 ^a^	1.00 ± 0.15 ^a^	1.00 ± 0.42	1.00 ± 0.15	1.00 ± 0.12 ^d^	1.00 ± 0.37	1.00 ± 0.49
CA	0.28 ± 0.06 ^b^	0.14 ± 0.04 ^b^	0.15 ± 0.08 ^c^	0.32 ± 0.34	7.22 ± 1.63	1.52 ± 0.21 ^b^	0.97 ± 0.65	0.99 ± 0.97
VC	0.26 ± 0.08 ^b^	0.18 ± 0.02 ^b^	0.46 ± 0.14 ^b^	1.01 ± 0.22	3.78 ± 1.47	1.25 ± 0.03 ^c^	0.90 ± 0.61	0.40 ± 0.26
CA + VC	0.12 ± 0.05 ^c^	0.17 ± 0.04 ^b^	0.25 ± 0.02 ^c^	0.23 ± 0.11	10.56 ± 2.50	3.29 ± 0.18 ^a^	0.35 ± 0.18	0.36 ± 0.13
cinnamaldehyde								
-	0.63 ± 0.41	0.59 ± 0.44	0.73 ± 0.31	1.01 ± 0.32 ^a^	2.39 ± 1.76 ^b^	1.12 ± 0.15	0.95 ± 0.49	0.70 ± 0.49
+	0.20 ± 0.10	0.15 ± 0.04	0.20 ± 0.08	0.27 ± 0.25 ^b^	8.89 ± 2.66 ^a^	2.41 ± 0.94	0.66 ± 0.56	0.68 ± 0.74
vitamin C								
-	0.64 ± 0.40	0.57 ± 0.46	0.57 ± 0.46	0.66 ± 0.51	4.11 ± 3.43 ^b^	1.26 ± 0.32	0.98 ± 0.50	1.00 ± 0.74 ^a^
+	0.19 ± 0.10	0.17 ± 0.03	0.36 ± 0.15	0.62 ± 0.44	7.17 ± 4.05 ^a^	2.27 ± 1.08	0.63 ± 0.52	0.38 ± 0.20 ^b^
*p*-value								
cinnamaldehyde	<0.001	<0.001	<0.001	<0.001	<0.001	<0.001	0.163	0.919
vitamin C	<0.001	<0.001	<0.001	0.756	<0.001	<0.001	0.089	0.015
interaction	<0.001	<0.001	<0.001	0.676	0.682	<0.001	0.212	0.952
	42 d							
CON	1.00 ± 0.17 ^a^	1.00 ± 0.14 ^a^	1.00 ± 0.20 ^a^	1.00 ± 0.13 ^a^	1.00 ± 0.23 ^d^	1.00 ± 0.22	1.00 ± 0.42 ^b^	1.00 ± 0.50
CA	0.51 ± 0.27 ^b^	0.16 ± 0.10 ^c^	0.55 ± 0.19 ^b^	0.27 ± 0.08 ^c^	18.34 ± 4.33 ^b^	1.79 ± 0.73	0.56 ± 0.09 ^c^	0.69 ± 0.22
VC	0.42 ± 0.23 ^b^	0.46 ± 0.14 ^b^	0.45 ± 0.11 ^b^	0.37 ± 0.06 ^c^	4.03 ± 1.41 ^c^	1.86 ± 0.87	1.32 ± 0.30 ^b^	3.05 ± 1.19
CA + VC	0.50 ± 0.26 ^b^	0.23 ± 0.07 ^c^	0.52 ± 0.19 ^b^	0.54 ± 0.16 ^b^	27.30 ± 4.18 ^a^	2.86 ± 0.79	2.83 ± 0.42 ^a^	1.93 ± 0.74
cinnamaldehyde								
-	0.71 ± 0.36	0.73 ± 0.32	0.73 ± 0.32	0.68 ± 0.34	2.52 ± 1.85	1.43 ± 0.75 ^b^	1.16 ± 0.38	2.02 ± 1.38 ^a^
+	0.50 ± 0.25	0.20 ± 0.09	0.54 ± 0.18	0.41 ± 0.19	22.82 ± 6.19	2.32 ± 0.92 ^a^	1.69 ± 1.22	1.31 ± 0.83 ^b^
vitamin C								
-	0.76 ± 0.33	0.58 ± 0.45	0.77 ± 0.30	0.64 ± 0.39	9.67 ± 9.51	1.39 ± 0.66 ^b^	0.78 ± 0.37	0.84 ± 0.40 ^b^
+	0.46 ± 0.24	0.34 ± 0.16	0.49 ± 0.15	0.46 ± 0.15	15.67 ± 12.51	2.36 ± 0.95 ^a^	2.08 ± 0.86	2.49 ± 1.11 ^a^
*p*-value								
cinnamaldehyde	0.045	<0.001	0.016	<0.001	<0.001	0.005	0.001	0.031
vitamin C	0.005	<0.001	0.001	0.001	<0.001	0.003	<0.001	<0.001
interaction	0.008	<0.001	0.002	<0.001	0.029	0.705	<0.001	0.206

Abbreviations: CON, basal diet; CA, basal diet with 300 g/t of cinnamaldehyde; VC, basal diet with 300 g/t of vitamin C; CA + VC, basal diet with 300 g/t of cinnamaldehyde and 300 g/t of vitamin C in combination. Different letters in the same column indicate significant differences (*p* < 0.05), and the same letter means no significant difference (*p* > 0.05). The main effects were compared in the case of no-significant interaction effects. “+” represent to add, “-” represents no added. ^1^ IL-10: interleukin-10. ^2^ IL-6: interleukin-6. ^3^ TNF-α: tumor necrosis factor α. ^4^ IL-1β: interleukin-1β. ^5^ NF-κB: nuclear factor kappa B. ^6^ MyD-88: myeloid differentiation factor88. ^7^ TLR-4: toll-like receptor-4. ^8^ TLR-2: toll-like receptor-2.

**Table 8:** Ingredients and composition of the basal experimental diets.

Ingredients, %	Starter Diet	Finisher Diet
Corn	9.40	45.30
Wheat	20.00	20.00
Broken rice	30.00	0.00
Corn gluten meal	0.00	6.00
Soybean meal	31.00	13.50
Cottonseed meal	3.00	6.00
Hydrolyzed feather meal	1.00	1.50
Soybean oil	1.20	3.30
Dicalcium phosphate	1.30	1.05
Limestone	1.30	1.40
Salt	0.20	0.20
Trace mineral premix ^1^	0.20	0.20
Vitamin premix ^2^	0.03	0.03
Choline chloride (50%)	0.16	0.16
Phytase	0.02	0.02
L-Lysine sulfate (65%)	0.38	0.58
DL-Methionine (98%)	0.28	0.17
L-Threonine (98.5%)	0.15	0.12
L-Arginine (98%)	0.02	0.00
L-Arginine sulfate (98%)	0.00	0.12
L-Valine (98%)	0.04	0.00
L-Isoleucine (90%)	0.00	0.10
Sodium bicarbonate	0.25	0.25
Nutrient composition, % ^3^		
ME (kcal/kg)	2930	3150
CP, %	22.06	20.10
Lysine, %	1.20	1.15
Methionine, %	0.54	0.48
Methionine + Cysteine, %	0.87	0.89
Threonine, %	0.79	0.90
Calcium, %	0.94	0.86
NPP, %	0.37	0.30

^1^ The trace mineral premix provided the following per kg of diets: Cu, 16 mg (as CuSO_4_·5H_2_O); Zn, 110 mg (as ZnSO_4_); Fe, 80 mg (as FeSO_4_·H_2_O); Mn, 120 mg (as MnO); Se, 0.3 mg (as Na_2_SeO_3_); I, 1.5 mg (as KI); Co, 0.5 mg. ^2^ The vitamin premix provided the following per kg of diets: vitamin A, 10 000 IU; vitamin D_3_, 2400 IU; vitamin E, 20 mg; vitamin K_3_, 2 mg; vitamin B_1_, 2 mg; vitamin B_2_, 6.4 mg; VB_6_, 3 mg; VB_12_, 0.02 mg; biotin, 0.1 mg; folic acid, 1 mg; pantothenic acid, 10 mg; nicotinamide, 30 mg. ^3^ All the nutrient levels are calculated values.

**Table 9:** Primers used in real-time quantitative PCR.

Gene	Gene Bank ID	Primer Sequence (5′-3′)
β-actin	XM_027015741.1	F: CAACACAGTGCTGTCTGGTGGTAC R: CTCCTGCTTGCTGATCCACATCTG
Occludin	D21837.1	F: ACGGCAGCACCTACCTCAA R: GGGCGAAGAAGCAGATGAG
Claudin-1	AY750897.1	F: CATACTCCTGGGTCTGGTTGGT R: GACAGCCATCCGCATCTTCT
ZO-1 ^1^	XM_413773	F: CTTCAGGTGTTTCTCTTCCTCCTC R: CTGTGGTTTCATGGCTGGATC
Mucin-2	NM_001318434.1	F: TCACCCTGCATGGATACTTGCTCA R: TGTCCATCTGCCTGAATCACAGGT
GPX-1 ^2^	HM_590226.1	F: ACGGCGCATCTTCCAAAG R: TGTTCCCCCAACCATTTCTC
CAT ^3^	NM_001031215.2	F: AGACATCTTCGCTGTGGTGA R: CGAGGATGTTGATGCAGGTG
SOD1 ^4^	NM_205064.1	F: GGTGCTCACTTTAATCCTG R: CTACTTCTGCCACTCCTCC
IL-10 ^5^	NM_001004414.3	F: CAGACCAGCACCAGTCATCAG R: ATCCCGTTCTCATCCATCTTCTCG
IL-6 ^6^	NM_204628.1	F: CAAGAAGTTCACCGTGTGCG R: GGAGAGCTTCGTCAGGCATT
TNF-α ^7^	NM_204267.2	F: CCTGCTGGGGGAATGCTAGG R: AGCGTTGTCTGCTCTGTAGC
IL-1β ^8^	NM_204524.1	F: TCTGCCTGCAGAAGAAGCC R: CTCCGCAGCAGTTTGGTCAT
NF-κB ^9^	NM_205129.1	F: ACCCCTTCAATGTGCCAATG R: TCAGCCCAGAAACGAACCTC
MyD88 ^10^	NM_001030962.3	F: TGCAAGACCATGAAGAACGA R: TCACGGCAGCAAGAGAGATT
TLR-4 ^11^	NM_001030693.1	F: GGATCTTTCAAGGTGCCACA R: CAAGTGTCCGATGGGTAGGT
TLR-2 ^12^	NM_003264.3	F: TAATACGACTCACTATAGGG R: TAGAAGGCACAGTCGAGG

Abbreviations: F, forward; R, reverse. ^1^ ZO-1: Zonula Occludens-1. ^2^ GPX-1: glutathione peroxidase 1. ^3^ CAT: catalase. ^4^ SOD1: superoxide dismutase 1. ^5^ IL-10: interleukin-10. ^6^ IL-6: interleukin-6. ^7^ TNF-α: tumor necrosis factor α. ^8^ IL-1β: interleukin-1β. ^9^ NF-κB: nuclear factor kappa B. ^10^ MyD-88: myeloid differentiation factor88. ^11^ TLR-4: toll-like receptor-4. ^12^ TLR-2: toll-like receptor-2.

## Data Availability

Data is contained within the article.
